# Cytochrome c oxidase is activated by the oncoprotein Ras and is required for A549 lung adenocarcinoma growth

**DOI:** 10.1186/1476-4598-11-60

**Published:** 2012-08-23

**Authors:** Sucheta Telang, Kristin K Nelson, Deanna L Siow, Abdullah Yalcin, Joshua M Thornburg, Yoannis Imbert-Fernandez, Alden C Klarer, Hanan Farghaly, Brian F Clem, John W Eaton, Jason Chesney

**Affiliations:** 1 Molecular Targets Program, James Graham Brown Cancer Center, University of Louisville, Louisville, KY, USA; 2 Department of Pathology, University of Louisville, Louisville, KY, USA

**Keywords:** Mitochondria, Glycolysis, Tricarboxylic acid cycle, Electron transport chain, Oncogene

## Abstract

**Background:**

Constitutive activation of Ras in immortalized bronchial epithelial cells increases electron transport chain activity, oxygen consumption and tricarboxylic acid cycling through unknown mechanisms. We hypothesized that members of the Ras family may stimulate respiration by enhancing the expression of the Vb regulatory subunit of cytochrome *c* oxidase (COX).

**Results:**

We found that the introduction of activated H-Ras^V12^ into immortalized human bronchial epithelial cells increased eIF4E-dependent COX Vb protein expression simultaneously with an increase in COX activity and oxygen consumption. In support of the regulation of COX Vb expression by the Ras family, we also found that selective siRNA-mediated inhibition of K-Ras expression in A549 lung adenocarcinoma cells reduced COX Vb protein expression, COX activity, oxygen consumption and the steady-state concentration of ATP. We postulated that COX Vb-mediated activation of COX activity may be required for the anchorage-independent growth of A549 cells as soft agar colonies or as lung xenografts. We transfected the A549 cells with COX Vb small interfering or shRNA and observed a significant reduction of their COX activity, oxygen consumption, ATP and ability to grow in soft agar and as poorly differentiated tumors in athymic mice.

**Conclusion:**

Taken together, our findings indicate that the activation of Ras increases COX activity and mitochondrial respiration in part via up-regulation of COX Vb and that this regulatory subunit of COX may have utility as a Ras effector target for the development of anti-neoplastic agents.

## Background

Activating mutations of the Ras guanosine nucleotide-binding proteins cause insensitivity to GTPase-activating proteins (GAP) and are common genetic alterations of human cancers [1]. Activated GTP-bound Ras family members promote survival and proliferation in part by increasing glucose uptake and flux into the synthesis of ribose-5-phosphate and lactate [2-4]. Recently, constitutive activation of Ras also has been found to increase mitochondrial tricarboxylic acid (TCA) cycle activity and oxygen consumption and to sensitize cells to the ATP-depleting and cytotoxic effects of electron transport perturbation using the complex I inhibitor rotenone [5]. These data suggested that activation of the Ras signalling pathway in immortalized cells might increase reliance on mitochondrial metabolism for energy and anabolism [6]. In support of this conclusion, disruption of mitochondrial function caused by loss of the mitochondrial transcription factor A (TFAM) gene reduced tumorigenesis in an oncogenic K-Ras-driven mouse model of lung cancer [7]. Additionally, elevated glycolytic flux into multiple mitochondrial TCA cycle products has been observed in human lung tumors *in vivo*[8] and these tumors commonly express mutated Ras [9]. Last, several agents that selectively disrupt mitochondrial function (*termed* mitocans) have been found to interfere with the bioenergetic functions of cancer cells and suppress tumor growth [10,11]*.* Understanding the precise mechanisms whereby Ras family members regulate mitochondrial metabolism and the functional importance of this metabolic effect for the tumor-forming potential of Ras-transformed cells may yield new molecular targets for the development of anti-cancer agents.

The mitochondrial electron transport chain consists of four respiratory enzyme complexes that serve to create a proton gradient [12]. Cytochrome *c* oxidase (COX), or complex IV, is the terminal enzyme in the electron transport chain which pumps protons from the matrix into the intermembrane space forming an electrochemical gradient across the inner mitochondrial membrane to generate ATP [13]. COX functions to catalyze electron transfer from cytochrome *c* to molecular oxygen via four redox centers [14]. Although COX only has ~20% total control over ATP synthesis [15], studies *in vivo* have indicated that COX represents the rate-limiting step of the electron transport chain [6]. COX is composed of thirteen different subunits [16,17] - subunits I-III are encoded by the mitochondrial genome whereas the other ten subunits are encoded by nuclear genes [18]. The nuclear subunits are synthesized as precursor proteins and imported into the mitochondria where the complex is assembled [19]. Within the thirteen subunit complex, the nuclear-encoded subunit Vb serves a unique role in the regulation of COX activity as this particular subunit faces the matrix side and is sufficient to increase both COX activity and oxygen consumption in HeLa cervical adenocarcinoma cells [20]. Interestingly, the expression of subunit Vb has been found to be increased in neoplastic cells and to positively correlate with the progression of cells from normal epithelia to invasive carcinoma [21]. These observations suggest that COX Vb not only may serve as an essential regulatory subunit of COX but also may be mediating the increase in mitochondrial metabolism caused by activation of Ras [5].

In the current study, we demonstrate that introduction of the activated H-Ras^V12^ GTPase into immortalized bronchial epithelial cells increases COX Vb protein expression, COX activity and oxygen consumption and that COX Vb protein expression is required to maintain the high oxygen consumption and ATP concentration of A549 lung adenocarcinoma cells that express oncogenic K-Ras. We also find that selective COX Vb inhibition markedly inhibits soft agar colony formation and poorly differentiated A549 xenograft growth in mice without affecting proliferation *in vitro*. Taken together, these observations suggest that COX may be an appropriate molecular target for the development of anti-neoplastic agents.

## Results

### H-Ras^V12^ increases COX Vb protein expression and activity

**Figure 1:**
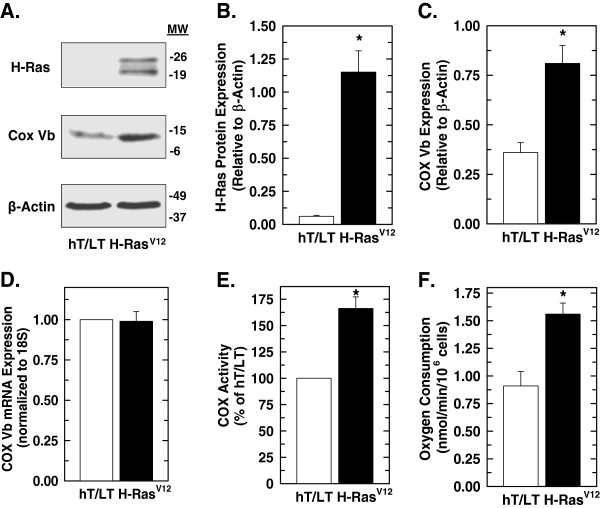
**Introduction of oncogenic H-Ras**^**V12 **^**into immortalized human bronchial epithelial cells increases COX Vb protein expression, COX activity and oxygen consumption.** hT/LT and H-Ras^V12^ cells were examined for H-Ras and COX Vb protein expression by Western blot analysis (**A**) and COX Vb mRNA expression by real-time RT-PCR (**D**). Densitometry of H-Ras (**B**) and COX Vb (**C**) protein expression relative to β-Actin expression was determined. Enzyme activity assays of COX (**E**) and oxygen consumption (**F**) were performed as described. Data are expressed as the mean ± SD of three experiments. *p value < 0.05 compared to hT/LT.

To explore the potential regulation of COX by Ras, we employed normal human bronchial epithelial cells that had been immortalized with the telomerase catalytic subunit and SV40 large T antigen (hT/LT cells) and then transformed with H-Ras^V12^ (H-Ras^V12^) [22]. Initially, we analyzed the hT/LT cells and H-Ras^V12^ cells for H-Ras protein expression by Western blot analysis and observed a marked increase in both wild-type H-Ras (lower band) and H-Ras^V12^ which displays reduced electrophoretic mobility [23] (upper band) (Figure 1A, B). These data suggest that introduction of H-Ras^V12^ may activate a positive feedback loop that leads to increased wild-type H-Ras expression. We then examined the cells for COX Vb mRNA expression by real-time RT-PCR and for protein expression by Western blot and observed no increase in COX Vb mRNA (Figure 1D) but an increase in COX Vb protein expression with the introduction of H-Ras^V12^ (Figure 1A, C). The introduction of H-Ras^V12^ into the hT/LT cells simultaneously increased COX activity (Figure 1E) and oxygen consumption (Figure 1F). Given that the only genetic difference between the immortalized hT/LT and H-Ras^V12^-transformed cells is the ectopic expression of H-Ras^V12^, these results indicate that activation of Ras increases or stabilizes protein expression of COX Vb, stimulates COX activity and increases oxygen consumption.

### COX Vb protein expression is dependent on eukaryotic translation initiation factor 4E (eIF4E) expression

**Figure 2:**
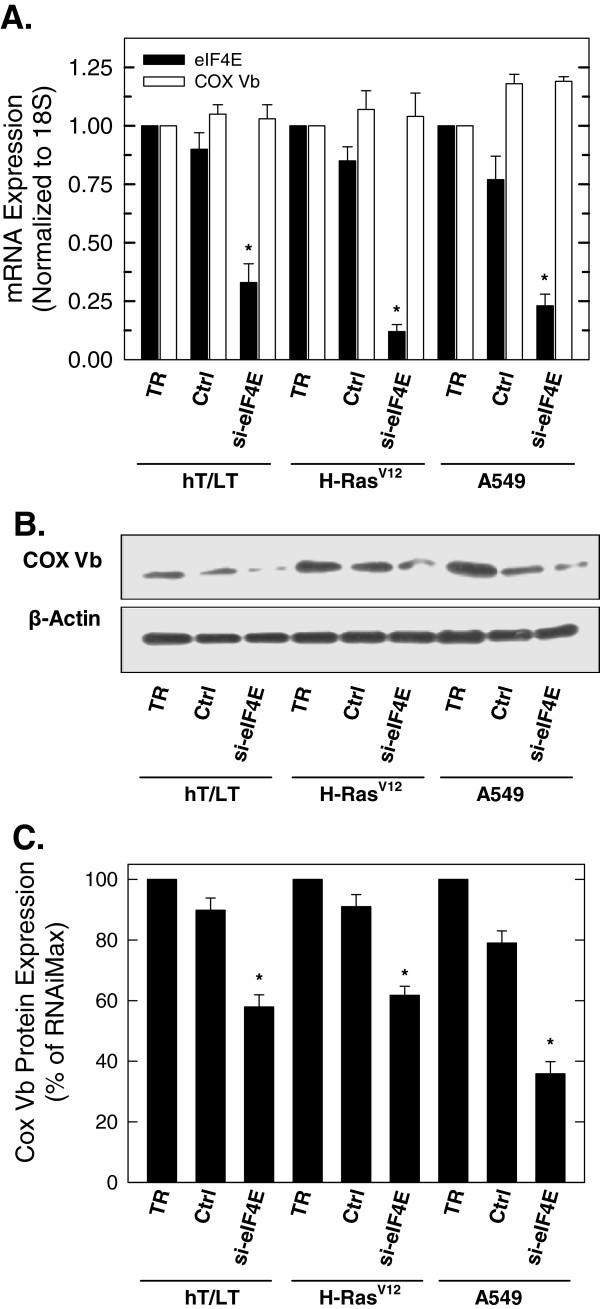
**Selective inhibition of eIF4E reduces COX Vb protein expression but has no effect on β-Actin protein expression.** hT/LT, H-Ras^V12^ and A549 cells were transfected with transfection reagent alone, control siRNA or siRNA specific for eIF4E for 48 hours and then examined for eIF4E and COX Vb mRNA expression by real-time RT-PCR (**A**) and COX Vb and β-actin protein expression using Western blot analysis (**B**). Protein expression was quantified by densitometric analyses (**C**) and expressed as percentage of transfection reagent. Data are expressed as the mean ± SD of three experiments. * *p* value <0.05.

Since H-Ras^V12^ expression had no effect on COX Vb mRNA but caused an increase in COX Vb protein expression, we postulated that H-Ras^V12^ may increase the efficiency of COX Vb mRNA translation. Activation of Ras signalling can increase the binding of the Eukaryotic Translation Initiation Factor 4E (eIF4E) to multiple mRNAs either through MAPK activation of Mnk which directly phosphorylates eIF4E or through the PI3K/AKT/mTOR-mediated phosphorylation and inhibition of inhibitory 4E binding proteins [24]. Although an increase in eIF4E binding to the 5´,7-methylguanosine cap structure of mRNAs stimulates the translation of all cap-dependent mRNAs, lengthy, GC-rich and highly structured 5´ UTRs can markedly reduce efficient RNA unwinding by eIF4E. COX Vb has a long (266 bp), GC-rich (56%) 5’-UTR and we thus examined the consequences of selective siRNA-mediated eIF4E inhibition on the induction of COX Vb protein expression caused by H-Ras^V12^ (Figure 2A-C). Whereas silencing of eIF4E mRNA expression had no effect on COX Vb mRNA expression (Figure 2A), COX Vb protein expression in the hT/LT-immortalized, H-Ras^V12^-transformed cells and K-Ras^S12^ positive A549 lung adenocarcinoma cells was reduced by transfection with the eIF4E siRNA species (Figure 2B, C). Importantly, eIF4E siRNA transfection had no effect on β-actin protein expression, which contains a short non-complex 5’-UTR, in any of the three cell types examined (Figure 2B, C). These data indicate that COX Vb protein expression is dependent on eIF4E activity and that the observed increase in COX Vb caused by introduction of oncogenic Ras can be partially attenuated by selective inhibition of eIF4E.

### Selective inhibition of K-Ras suppresses COX Vb activity, oxygen consumption and intracellular ATP in A549 cells

**Figure 3:**
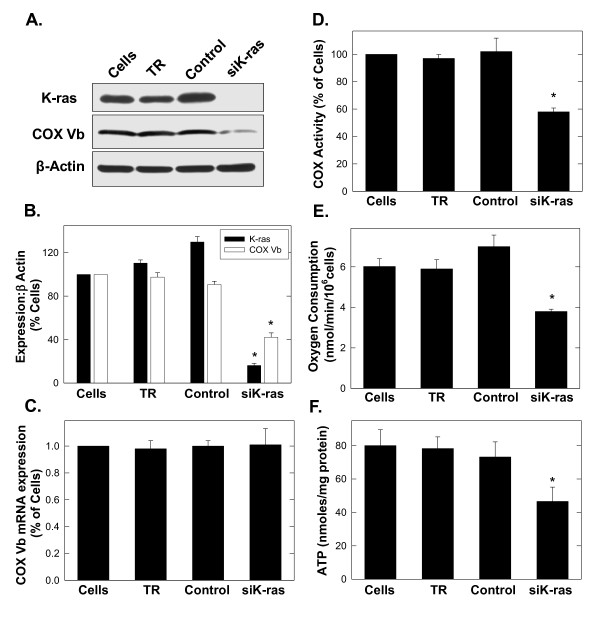
**Selective inhibition of K-Ras expression by A549 lung adenocarcinoma cells reduces COX activity, oxygen consumption and ATP.** A549 cells were transfected with transfection reagent (TR) alone, control siRNA or siRNA specific for K-Ras for 72 hours and then examined for COX Vb protein expression (**A** and **B**), COX Vb mRNA expression (**C**), COX activity (**D**), oxygen consumption (**E**) and ATP (**F**). Data are expressed as the mean ± SD of three experiments. * *p* value <0.05.

We next selectively inhibited the expression of K-Ras in A549 cells using siRNA and examined the consequences on COX Vb protein expression, COX activity, oxygen consumption and ATP concentration. Transfection of A549 cells with K-Ras siRNA caused a reproducible reduction in COX Vb protein expression (Figure 3A, B) without affecting COX Vb mRNA expression (mRNA fold change of K-Ras siRNA relative to control siRNA = 1.01 ± 0.12) (Figure 3C).

Selective K-Ras inhibition also reduced COX activity, oxygen consumption and the steady-state concentration of ATP in the A549 cells (Figure 3D-F). These data coupled to the observations that ectopic expression of H-Ras^V12^ increases COX Vb expression and COX activity further support the conclusion that COX Vb expression is regulated by Ras signalling.

### Suppression of COX Vb expression decreases anchorage-independent growth as soft agar colonies

**Figure 4:**
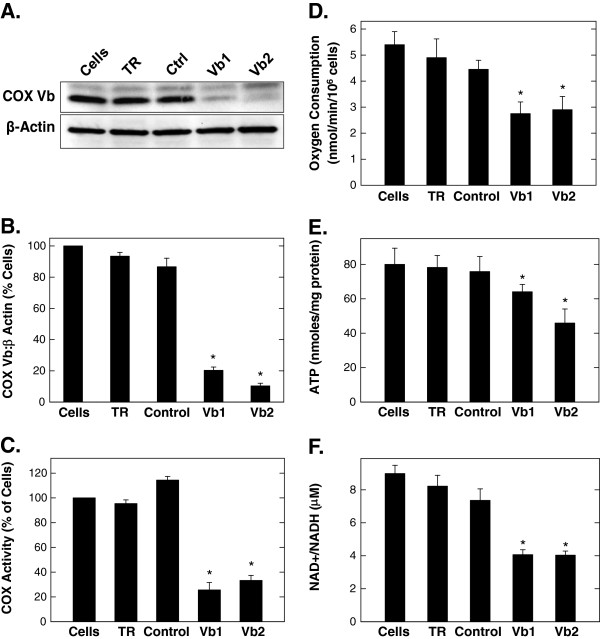
**Selective inhibition of COX Vb by A549 cells reduces COX activity, oxygen consumption and ATP.** A549 cells were transfected with transfection reagent alone, control siRNA or siRNA specific for the ORF (Vb1) or 3’-UTR (Vb2) of COX Vb for 48 hours and then examined for COX Vb protein expression (**A** and **B**), COX activity (**C**), oxygen consumption (**D**), ATP (**E**) and NAD+/NADH ratios (**F**). Data are expressed as the mean ± SD of three experiments. * *p* value <0.05.

**Figure 5:**
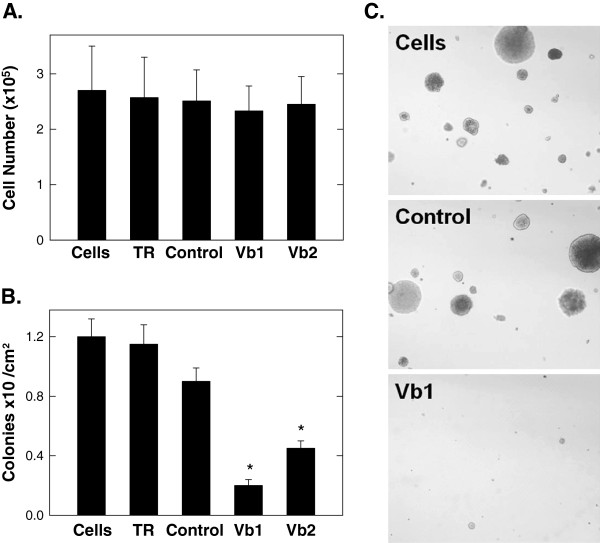
**Selective inhibition of COX Vb expression by A549 cells suppresses anchorage-independent growth in soft agar. A**. 10^5^ A549 cells transfected with COX Vb or control siRNA were cultured for 48 hours and viable cells were enumerated using light microscopy. **B**. After the 48 hour transfection, A549 cells were plated on 6 cm dishes containing DMEM with 0.6% agar. Cells were fed with 0.2% agar in media every three days. After 21 days, soft agar colonies were enumerated. **C**. Representative photomicrographs of the soft agar colonies. Data are expressed as the mean ± SD of five (A) and three (B) experiments. * *p* value <0.05.

In order to directly examine the hypothesis that COX Vb expression is required for tumor growth, we next transiently transfected the A549 cells with two COX Vb siRNA species specific for the open reading frame (Vb1) or for the 3’-untranslated region (Vb2). Forty eight hours after transfection, Western blot analyses indicated that both COX Vb siRNA species inhibited the protein expression of COX Vb (Figure 4A, B). We found that transfection of A549 cells with either COX Vb siRNA decreased COX activity, oxygen consumption, intracellular ATP and the NAD+/NADH ratio compared to controls (cells, transfection reagent alone or control siRNA) (Figures 4C-F). We next sought to assess the consequences of reduced mitochondrial metabolism on anchorage-independent survival and growth in soft agar. We observed no significant decrease in A549 anchorage-dependent cell proliferation 48 hours after transfection with COX Vb-specific siRNA molecules (Figure 5A) but found that the siRNA-mediated silencing of COX Vb markedly reduced subsequent A549 soft agar colony formation and growth (Figure 5B, C). Although the precise metabolic explanation for the apparent sensitivity of soft agar colony growth to COX Vb inhibition is not well understood, nutrient and oxygen diffusion limitations imposed by adjacent cells within the colony combined with reduced COX activity may cause synergistic cytostasis.

### Suppression of COX Vb expression decreases anchorage-independent growth as tumors in athymic mice

**Figure 6:**
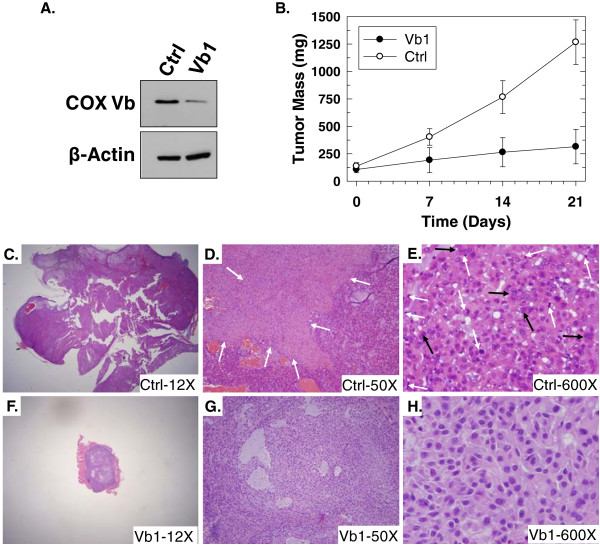
**Stable COX Vb shRNA expression in A549 cells reduces the outgrowth of A549 xenografts and inhibits multiple histopathological indicators of poor differentiation in athymic mice.** 5x10^6^ A549 cells stably transfected with control or COX Vb-specific shRNA were subjected to Western blot analysis for COX Vb or β-actin expression (**A**) and then injected subcutaneously into athymic mice. After 4 weeks, tumors were measured weekly for a total of 3 additional weeks (timepoints 0, 7, 14 and 21) using microcalipers. Tumor mass was calculated based on bidimensional measurements and data are expressed as the mean ± SEM of two experiments (**B**). After 7 weeks of growth, mice were euthanized and tumors were excised, fixed in formalin, paraffin-embedded, sectioned and stained with hematoxylin/eosin. Light micrographs demonstrate that, relative to the COX Vb1 shRNA-transfected A549 tumors (**F**-**H**), the control A549 tumors were higher grade, poorly differentiated and invasive (**C**-**E**), contain large areas of tumor necrosis (D; white arrows), pleomorphic nuclei (E, black arrows), and numerous mitotic figures (E, white arrows).

The ability to grow as soft agar colonies can be predictive of tumor-forming capacity in athymic mice. We subcutaneously injected 5 × 10^6^ live A549 cells stably transfected with a plasmid expressing the COX Vb1 shRNA or a control shRNA and measured tumor outgrowth using microcalipers. Suppression of COX Vb protein expression in the COX Vb1 shRNA clone was confirmed by Western blot (Figure 6A). Although both groups of mice had detectable subcutaneous A549 tumors after 4 weeks, the outgrowth of the COX Vb1 shRNA expressing tumors was markedly reduced over the following 3 weeks (Figure 6B). We then euthanized the mice, excised the tumors and stained them with Hematoxylin and Eosin (Figure 6C-H). We found that the control A549 tumors contained large areas of tumor necrosis (see white arrows, Figure 6D), pleomorphic nuclei (black arrows, Figure 6E) and numerous mitotic figures (white arrows, Figure 6E), all of which are consistent with high-grade, rapidly growing but poorly differentiated adenocarcinomas. In contrast, we observed no tumor necrosis, low mitotic activity, and few pleomorphic nuclei in the COX Vb1 shRNA-transfected A549 tumors (Figure 6F-H), indicating that COX Vb inhibition in the A549 cells significantly lowered the histopathological grade of tumors *in vivo*. Given that COX Vb inhibition reduces oxygen consumption *in vitro*, we speculated that the oxygen concentration may be increased in the COX Vb shRNA A549 tumors relative to the control tumors. However, we examined the tumors for carbonic anhydrase IX protein expression, which is a target gene of HIF-1α, and observed no difference in expression (*data not shown*)*.*

## Discussion

In this study, we found that introduction of oncogenic H-Ras^V12^ into immortalized human bronchial epithelial cells enhanced eIF4E-dependent COX Vb protein expression, COX activity and oxygen consumption. Conversely, selective siRNA silencing of K-Ras expression in A549 cells was observed to selectively suppress COX Vb protein expression, COX activity and oxygen consumption. Transfection of the A549 cells with two independent siRNA molecules specific for COX Vb caused a marked decrease in COX activity, oxygen consumption, ATP, anchorage-independent soft agar colony growth and xenograft growth in athymic mice. Taken together, these data suggest that a metabolic regulatory pathway thus exists whereby Ras signalling activates eIF4E activity which, in turn, leads to increased COX Vb protein expression, COX activity and oxygen consumption. The reduction in xenograft growth that was caused by COX Vb inhibition was associated with reduced histological markers of high-grade poorly differentiated cells which may indicate that COX activity is required for the aggressiveness of certain lung adenocarcinomas. These observations support an examination of potential correlations between COX Vb protein expression by human lung adenocarcinomas and clinical outcomes including growth rates, response to therapy and overall survival. Additionally, these results indicate that COX may have utility as a target for the development of anti-cancer therapeutics, especially against tumors in which the Ras signalling pathway is activated.

Ras proto-oncogenes encode membrane-bound 21 kDa guanosine nucleotide-binding proteins that transduce mitogenic signals from tyrosine-kinase receptors. Whereas Ras mutations occur in ~30 percent of human cancers [9], activation of Ras signalling pathways (*e.g.* via epidermal growth factor receptor [EGFR] amplifications) is a nearly ubiquitous characteristic of human cancers. K-Ras^G12D^ transgenic mice develop tumors which express COX Vb [25], and activation of the Ras/cAMP/protein kinase A pathway in *Saccharomyces cerevisiae* increases COX activity and oxygen consumption [26]. Additionally, the ratio of COX Vb to COX I or COX II expression markedly increases during the progression from normal human prostate epithelium to invasive prostate carcinoma [21], a transformation that is dependent on the downstream Ras signalling pathways MAPK and PI3K/AKT [27]. The current study adds to this body of literature, demonstrating that the discrete introduction of the human oncogene H-Ras^V12^ into immortalized human bronchial epithelial cells not only increases protein expression of the regulatory Vb subunit but also oxygen consumption and COX activity. Perhaps more importantly, the current study demonstrates for the first time that COX Vb expression by Ras-transformed lung adenocarcinoma cells is required for their ability to grow as a high-grade, poorly differentiated tumor in athymic mice.

We previously reported that introduction of activated Ras into immortalized cells simultaneously increased glycolytic flux to lactate, TCA cycle activity and oxygen consumption [5]. Ras oncoproteins also directly increase the expression of uncoupling protein (UCP)-1, and UCP-2 expression has been found to be increased in most human colon adenocarcinomas in which up to 60% contain K-Ras mutations [28,29]. The mitochondrial TCA cycle and cytoplasmic GADPH reactions require oxidized NAD+ which is produced by LDH in the cytosol or by electron transport chain activity in the mitochondria. We postulate that Ras activation may cause simultaneous stimulation of COX activity and uncoupling of ATP synthase from the proton gradient in order to facilitate the continued oxidation of NADH to NAD+ necessary for glycolytic flux and TCA cycling [6].

Expression of oncogenic H-Ras^V12^ sensitizes immortalized cells to electron transport chain disruption with rotenone suggesting that activation of Ras signalling also may confer greater reliance on the electron transport chain for energy requirements [5]. Given that we have found that introduction of H-Ras^V12^ increases COX Vb protein expression and COX activity relative to immortalized cells, we speculate that the COX Vb subunit and the entire COX complex serve as essential metabolic downstream effectors of Ras. Dichloroacetate (DCA), an inhibitor of pyruvate dehydrogenase (PDH) kinase and thus a stimulator of PDH and flux into the TCA cycle, has been observed to increase oxygen consumption but to reduce tumor growth [30]. In contrast, we have found that selective inhibition of COX Vb reduced both oxygen consumption and tumor growth. Although the precise mechanism and rationale for these differences are not well understood, we postulate that the disruption of NADH oxidation caused by either the diversion of pyruvate away from LDH or by the inhibition of electron transport chain activity (Figure 4F) may yield a similarly deleterious effect on anabolic pathways such as the TCA cycle that are required for tumor growth.

## Conclusion

In conclusion, we find that activation of the Ras signalling pathway increases COX activity in part via eIF4E-dependent enhancement of COX Vb protein expression and that COX is required for tumorigenic growth. The continued characterization of the regulatory mechanisms and rationale for increased COX activity in transformed cells should allow us to tailor rational anti-metabolic approaches for the treatment of cancer.

## Methods

### Cell lines and culture

A549 human lung adenocarcinoma cells (American Type Culture Collection, Manassas, VA) were maintained in Dulbecco’s Modified Eagle Medium (DMEM, Invitrogen, Grand Island, NY) supplemented with 10% FCS and gentamicin sulfate (HyClone, Logan, UT). Normal human bronchial epithelial cells expressing telomerase and SV40 large T antigen (hT/LT) and activated Ras (H-Ras^V12^) were gifts from Dr. B. J. Rollins, Dana Farber Cancer Institute. hT/LT and H-Ras^V12^ cells were grown in BEGM medium formulated with SingleQuots (bovine pituitary extract, recombinant human epidermal growth factor, hydrocortisone, insulin, epinephrine, tri-iodothyronine, transferrin, gentamicin, amphotericin B and retinoic acid) (Lonza, Walkersville, MD). Media contained physiologic glucose (5.5 mM) and glutamine (1 mM), and cells were cultured under 21% O_2_ (normoxia) with 5% CO_2_ at 37°C (Heraeus).

### siRNA transfections

Cells were plated at 8 x 10^4^ cells/well in 6 well tissue culture plates and incubated at 5% CO_2_ and 37°C in DMEM as described above for 24 hours. Cells were then transfected with siRNA using RNAiMax (Invitrogen, Carlsbad, CA) according to the manufacturer’s protocol. For inhibition of COX Vb in A549 cells, two siRNA constructs were designed, one specific for the open reading frame region of COX Vb (COX Vb- ORF) and a second specific for the 3’ untranslated region (COX Vb- 3’UTR), termed COX Vb1 and COX Vb2 respectively. The target sequences (sense strands) were as follows: Vb1 = AGUAGGCUGCAUCUGUGAA and Vb2 = CAGUAAAGACUAGCCAUUG. siRNAs against lamin A/C and RNAiMax alone were included as controls. COX Vb siRNA duplexes were synthesized by Dharmacon (Lafayette, CO). For the inhibition of eIF4E, hT/LT, H-Ras^V12^ or A549 cells were transfected with pre-synthesized eIF4E siRNA (Ambion, Austin, TX). For the inhibition of K-Ras, A549 cells were transfected with pooled K-Ras siRNA (Santa Cruz Biotechnology, Santa Cruz, CA).

### Western blot analysis

Western blot analysis was carried out on protein lysates from A549, hT/LT, and H-Ras^V12^ cells transfected with siRNA as described above. Briefly, equal amounts of total protein (30 μg) from each sample were run on a 4-20% gradient SDS-PAGE gel under reducing conditions and proteins were transferred to PVDF membranes (BioRad, Hercules, CA). Membranes were probed with a 1:500 dilution of anti-COX Vb polyclonal mouse antibody (Invitrogen), a 1:1000 dilution of anti-H-Ras antibody or a 1:100 dilution of anti-K-Ras antibody (both from Santa Cruz). Immunoreactive proteins were visualized by incubating membranes with horseradish peroxidase-conjugated anti-mouse or anti-rabbit IgG antibody (1:10000) followed by reaction with ECL Plus (Amersham, St. Louis MO). Membranes were subsequently probed with a mouse monoclonal β-actin antibody (Sigma, St. Louis MO) as an internal protein loading control. Experiments were repeated four times. All data are expressed as the mean ± SD of three experiments. Statistical significance was assessed by the unpaired two-tail *T*-test.

### COX activity assay

COX activity was measured as described in Campian *et al.*[20]. Briefly, lysates of cells (at a concentration of 2 × 10^6^ cells/ml) were prepared in 0.25 M sucrose, 40 mM potassium chloride, 2 mM EGTA, 1 mg/ml bovine serum albumin, and 20 mM Tris–HCl (pH 7.2) and disrupted by 3 1 second bursts using a microtip Fisher model 100 sonic dismembrator at scale 3 (on a scale of 0–10 of the 100-watt maximum power output) (Fisher Scientific, Pittsburgh, PA). The lysate was centrifuged at 4000 × *g* for 10 min, the pellet discarded and the supernatant was used for COX assays. Assays contained 30 μg of protein and were performed at 37°C in 200 μl reaction volumes. The assay involved the addition of 40 μM ferrocytochrome *c* in an isosmotic medium (10 mM KH_2_PO_4_ (pH 6.5), 1 mg/ml bovine serum albumin, 0.3 M sucrose) containing 2.5 mM *n*-dodecyl maltoside to permeabilize mitochondrial membranes. The activity was calculated from the rate of decrease in absorbance of ferrocytochrome *c* at 550 nm (ε = 19.1 mM^–1^ cm^–1^). All data are expressed as the mean ± SD of three experiments. Statistical significance was assessed by the unpaired two-tail *T*-test.

### Oxygen consumption

Cells were detached with trypsin-EDTA and centrifuged at 1000 rpm for 5 minutes in complete culture medium. Oxygen consumption was measured using a Strathkelvin 782 Oxygen Meter (Strathkelvin Instruments, Glasgow, Scotland, UK). Respiration rates were measured using 1 × 10^6^ cells suspended in a total volume of 525 μL DMEM containing 10% FBS at 37°C for 10 minutes. A starting O_2_ concentration of 240 μM was assumed based on O_2_ solubility at conditions in this laboratory (1 atm/37°C). Experiments were repeated four times. The data shown are mean ± SD of three experiments. Statistical significance was assessed by the unpaired two-tail *T-*test.

### ATP assay

Cells were washed (while still adherent) with cold PBS x1, lysed with passive lysis buffer (1X, Molecular Probes, Invitrogen) added directly to the plates and immediately harvested by scraping. The lysates were flash frozen (to −80°C) and thawed (to 37°C) once to accomplish complete lysis and then centrifuged (at 4°C) for 30 seconds to clear the lysates. Intracellular ATP levels were determined using a bioluminescence assay (Molecular Probes) utilizing recombinant firefly luciferase and its substrate, D-luciferin. The luminescence was read in a TD-20/20 luminometer (Turner Designs, Sunnyvale, CA) at 560 nm. The ATP values were calculated using an ATP standard curve. The protein concentrations of the lysates were estimated using the bicinchoninic acid (BCA) assay (Pierce Biotechnology, Rockford, IL) and ATP was expressed as nmol per mg protein. Experiments were repeated five times. All data are expressed as the mean ± s.d. of three experiments. Statistical significance was assessed by the unpaired two-tail *T-*test.

### NAD+/NADH ratio assay

A549 cells treated with siRNA species for 48 hours were lifted using trypsin - EDTA, washed twice with cold PBS and 10^5^ cells were pelleted by centrifugation. NAD+ and NADH concentrations were measured using an enzyme-based assay (E2ND-100, Bioassay Systems, Hayward, CA). Briefly, the cell pellets were homogenized in either NAD+ extraction buffer (containing 0.40% HCl) or NADH extraction buffer (containing 0.40% NaOH), extracts heated to 60°C and then assay buffer (containing Tris(hydroxymethyl)aminomethane 3.0%, and BSA 0.10%) and extraction buffer (to neutralize the extracts) were added. Following centrifugation, supernatants were mixed with working reagent containing assay buffer, lactate dehydrogenase, diaphorase, lactate and tetrazolium dye (MTT) and optical density at 565 nm was recorded at time zero and at 15 minutes using a 96 well plate reader spectrophotometer. The difference in absorbance was compared with standard solutions and used to calculate NADH and NAD+ concentrations. All data are expressed as the mean ± s.d. of three experiments. Statistical significance was assessed by the unpaired two-tail *T*-test.

### Cell viability

For measurements of contact-dependent growth, cells were grown in appropriate media as described above. Following siRNA transfections, cells were grown for 48 h. Proliferation was determined using trypan blue exclusion. Cells were incubated in 20% trypan blue (Sigma) for 5 minutes. Cells excluding trypan blue were counted using a standard hemocytometer (Hausser Scientific) to determine the total number of viable cells. All data are expressed as the mean ± SD of five experiments. Statistical significance was assessed by the unpaired two-tail *T*-test.

### Soft agar colony formation assay

A549 cells with or without Vb siRNA transfection for 48 hours were plated at a density of 1 × 10^4^ cells/60-mm plate sandwiched by 3 ml bottom agar (0.6 %) and 3 ml top agar (0.3%). Cells were fed every three days by adding a new layer of top agar. After 21 days, colonies were counted in random 1 cm^2^ sections of each plate. All data are expressed as the mean ± SD of three experiments. Statistical significance was assessed by the unpaired two-tail *T*-test.

### Xenograft studies

A549 lung adenocarcinoma cells stably transfected with either COX Vb or a control shRNA were collected from exponential growth phase culture in DMEM supplemented with 10% FCS. Cells were washed twice and re-suspended in PBS (5 × 10^7^ cells/ml). Groups of CD1 female athymic mice (20 gm) were injected s.c. with 0.1 ml of the cell suspension (5 × 10^6^ cells). The tumors were followed from the time of appearance until 21 days. Tumor masses were determined in a blinded fashion with Vernier calipers according to the following formula: mass(mg) = (width, mm)^2^ × (length, mm)/2 [31]. All data are expressed as the mean ± SD of two experiments (n = 8 per group). Statistical significance was assessed by the unpaired two tail *T*-test. At the end of the experiment, mice were euthanized and the tumors were excised, fixed in formalin, embedded in paraffin and stained with Hematoxylin and Eosin or with anti-CA IX (Abcam, Cambridge, MA).

## Competing interests

The authors declare that they have no competing interests.

## Authors’ contributions

ST conducted the K-ras and COX Vb siRNA experiments, soft agar and *in vivo* experiments and participated in the conception of the manuscript. KKN assisted with the COX assay. DLS conducted the EIF4E siRNA experiments. AY assisted in the *in vivo* experiments. JMT assisted in the development of the COX assay. YI assisted in the *in vivo* experiments. ACK conducted the real time RT-PCR. HF conducted the histopathological review of the tumors. BFC assisted with the oxygen consumption experiments. JWE participated in the design of the experiments and interpretation of the data. JC conceived the research, directed all experiments and drafted the manuscript. All the authors have been involved in the drafting of the manuscript and have read and approved the final manuscript.
